# Quintuple Mental Foramina: A Rare Anatomical Variant Identified by High‐Resolution Cone Beam Computed Tomography—A Case Report

**DOI:** 10.1002/ccr3.73824

**Published:** 2026-11-04

**Authors:** Joul Kassis

**Affiliations:** ^1^ Department of Oral and Maxillofacial Surgery, Faculty of Dentistry Damascus University Damascus Syria; ^2^ Painfree Dentistry Sydney New South Wales Australia

**Keywords:** accessory mental foramina, anatomical variation, cone beam computed tomography, mandibular nerve injury, third molar extraction

## Abstract

This 26‐year‐old male presented with severe right mandibular pain. ALARA/ALADA‐compliant cone beam computed tomography revealed a rare bilateral quintuple mental foramina configuration: three on the right and two on the left. Third molar extraction proceeded without neurosensory complications. This report details the embryological, epidemiological, and surgical implications of this anatomical variation.

## Key Clinical Message

Standard anatomical assumptions can compromise patient safety. High‐resolution, ALARA/ALADA‐compliant cone beam computed tomography (CBCT) revealed an exceptionally rare bilateral quintuple mental foramina configuration. Preoperative 3D neurovascular mapping is essential to prevent neurosensory complications, avoid flap design inadvertencies, and address unexplained anesthetic failures during mandibular surgical interventions.

## 1 Introduction

The mental foramen (MF) is traditionally defined as a singular, circular or oval‐shaped osseous opening located on the buccal aspect of the mandibular body, typically situated in the alveolar process between or adjacent to the apices of the mandibular premolars. Anatomically, it serves as the terminal exit point for the mental nerve and its associated vessels, which branch from the inferior alveolar bundle. These structures supply sensory innervation to the lower lip, labial gingiva, and chin region, as well as essential vascularity to the surrounding soft and hard tissues [1].

Despite classical anatomical descriptions, the mandibular canal and its exits exhibit profound morphological diversity. Accessory mental foramina (AMF)—defined as any additional cortical openings communicating with the mandibular canal beyond the primary foramen—arise due to the premature branching of the mental nerve prior to exiting the bone during fetal development [2]. Rather than consolidating into a single nerve trunk, these nerve filaments navigate through discrete cortical micro‐channels. While a single unilateral AMF is a recognized clinical variant observed in approximately 2%–10% of various populations, the bilateral presence of multiple accessory foramina leading to a total of five mental foramina is an exceptionally rare anatomical phenomenon with minimal documentation in surgical literature [3].

The clinical and surgical significance of identifying these variations cannot be overstated. Standard diagnostic and operative protocols often rely on a “singular foramen” heuristic, which can lead to inadvertent neurovascular trauma, diagnostic errors, and procedural failure. As surgical interventions in the mandibular premolar and anterior regions such as apical surgery, implant placement, bone grafting, and orthognathic procedures become increasingly refined, understanding neurovascular exit variations has shifted from an academic curiosity to a mandatory prerequisite for patient safety.

This case report describes a quintuple mental foramina configuration (three on the right side, two on the left side), detailing the diagnostic rationale under radiation protection standards (ALARA/ALADA), discussing the epidemiological literature regarding sex and lateral predilection, and outlining the clinical management and postoperative follow‐up [4].

## 2 Case Report

A 26‐year‐old male presented to our clinic complaining of severe, radiating pain in the right posterior mandible, accompanied by localized buccal soft tissue swelling and mild trismus. The patient's medical history was unremarkable, with no history of facial trauma, mandibular fractures, or metabolic bone disease that could alter cortical bone morphology. Extra‐oral examination revealed mild facial asymmetry and palpable, tender right submandibular lymphadenopathy; trigeminal neurosensory function was intact bilaterally. Intraoral examination demonstrated a partially impacted mandibular right third molar with associated pericoronitis and tissue inflammation. A Written informed consent from the patient was obtained according to journal guidelines.

## 3 Radiographic Protocol & ALARA/ALADA Compliance

To properly evaluate the impacted third molar and assess complex regional anatomy prior to intervention, 3D imaging was indicated. In strict adherence to As Low As Reasonably Achievable (ALARA) and As Low As Diagnostically Acceptable (ALADA) radiation protection principles, a high‐resolution Cone Beam Computed Tomography (CBCT) scan (MyRay, Hyperion X9 Pro, Italy) was selected over conventional multi‐slice CT, using a field of view (FOV) optimized to the mandibular arch with a fine voxel size of 68 μm. This tailored protocol delivered the necessary high‐contrast spatial resolution to accurately trace neurovascular channels while minimizing radiation exposure to the patient.

The CBCT dataset was processed using iRYS viewer software and reconstructed into cross‐sectional, axial, coronal, sagittal, and 3D volumetric renders.

## 4 Detailed Morphological Analysis

Radiographic evaluation demonstrated a total of five distinct mental foramina distributed bilaterally: two on the left side of the mandible (Figure 1) and three on the right side (Figure 2).

**FIGURE 1:**
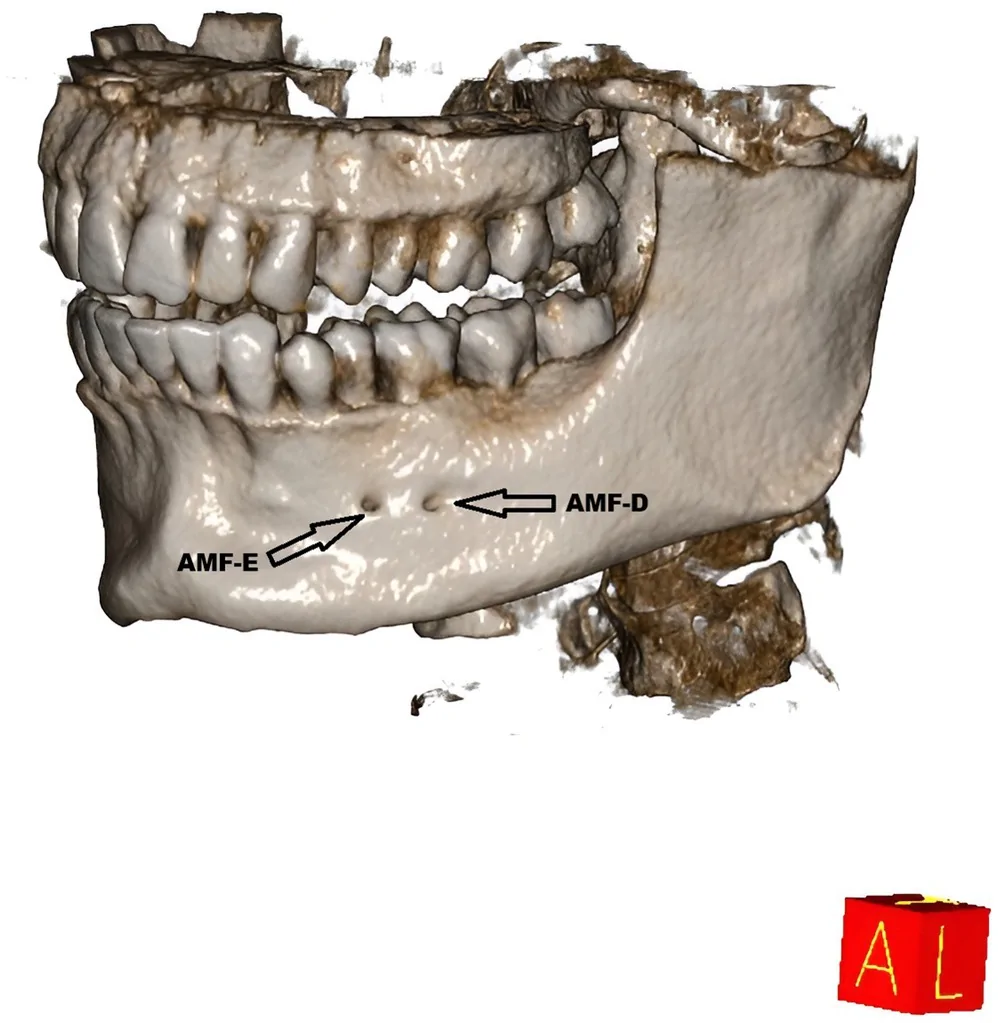
3D model of the CBCT imaging showing two mental foramina on the left side of the mandible: AMF‐D, AMF‐E.

**FIGURE 2:**
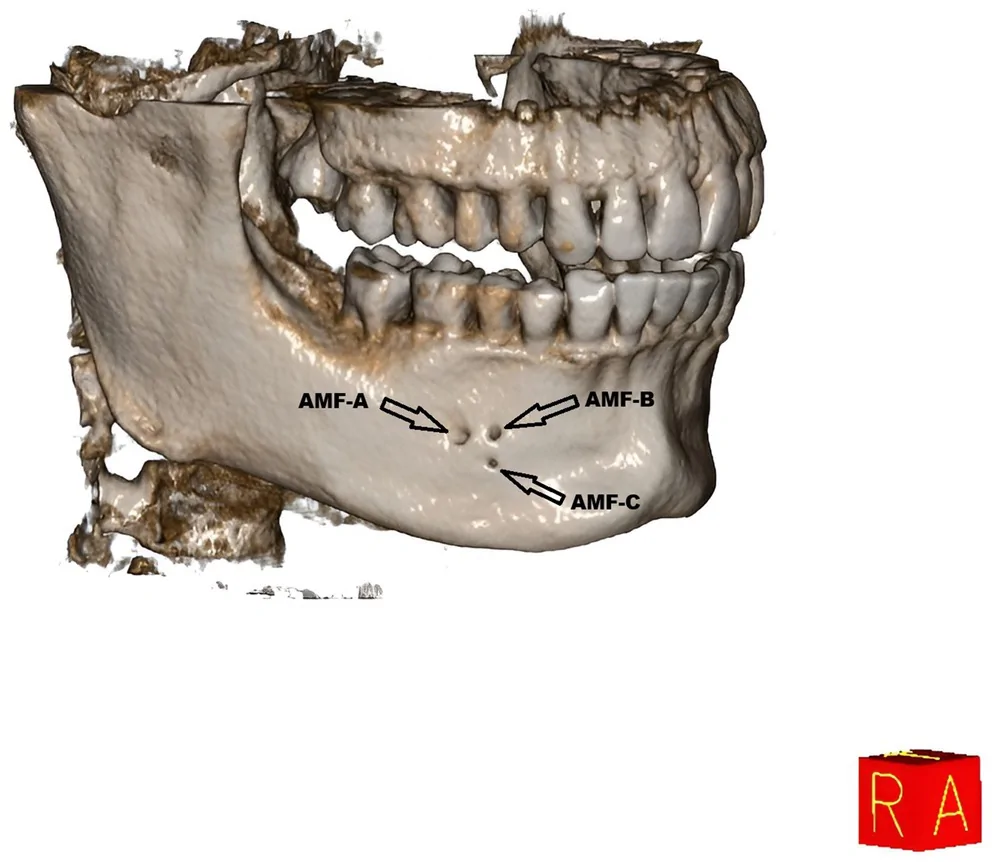
3D model of the CBCT imaging showing three mental foramina on the right side of the mandible, AMF‐A, AMF‐B, AMF‐C.

### 4.1 Right Mandibular Side (Three Foramina)

Right Primary Mental Foramen (MF1): Measuring 3.4 mm in maximum diameter, located vertically at the level of the second premolar apex and 11.0 mm superior to the inferior border of the mandible (Figure 3).

- Superior Posterior Accessory Foramen (AMF‐A): A 1.2 mm opening situated 4.0 mm superior and slightly posterior to MF1, positioned between the root apices of the second premolar and first molar (Figure 4A,B).
- Anterosuperior Accessory Foramen (AMF‐B): A 0.9 mm opening located anteriorly near the apex of the second premolar, representing the most anterior exit of the right cluster (Figure 4A,C).
- Inferior Accessory Foramen (AMF‐C): A 0.6 mm opening located 2 mm inferior to the AMF‐B represents the smallest foramen in this cluster (Figure 4A,C).

**FIGURE 3:**
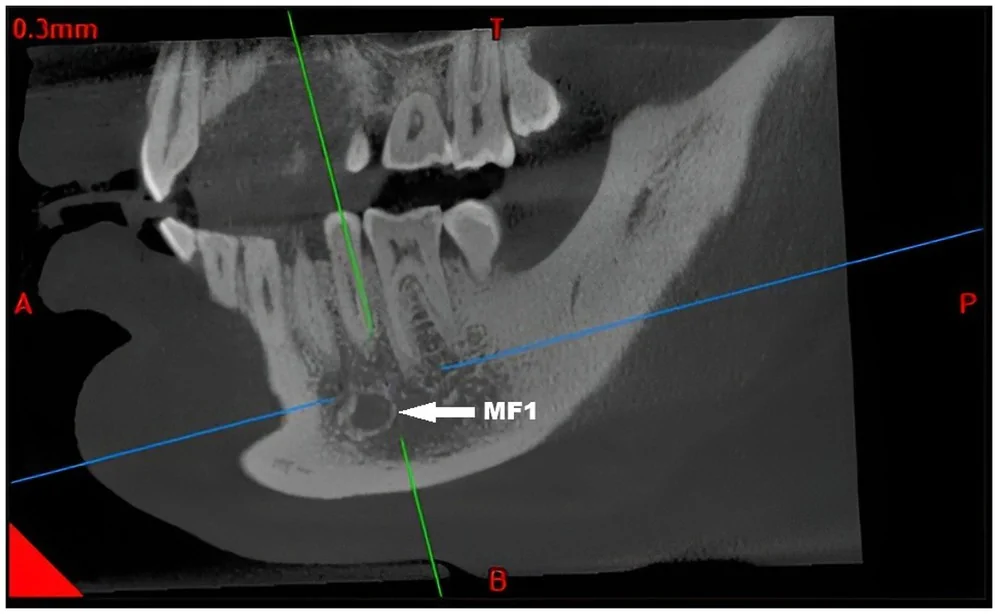
Sagittal radiographic view of the right mandible showing the main mental foramen MF1.

**FIGURE 4:**
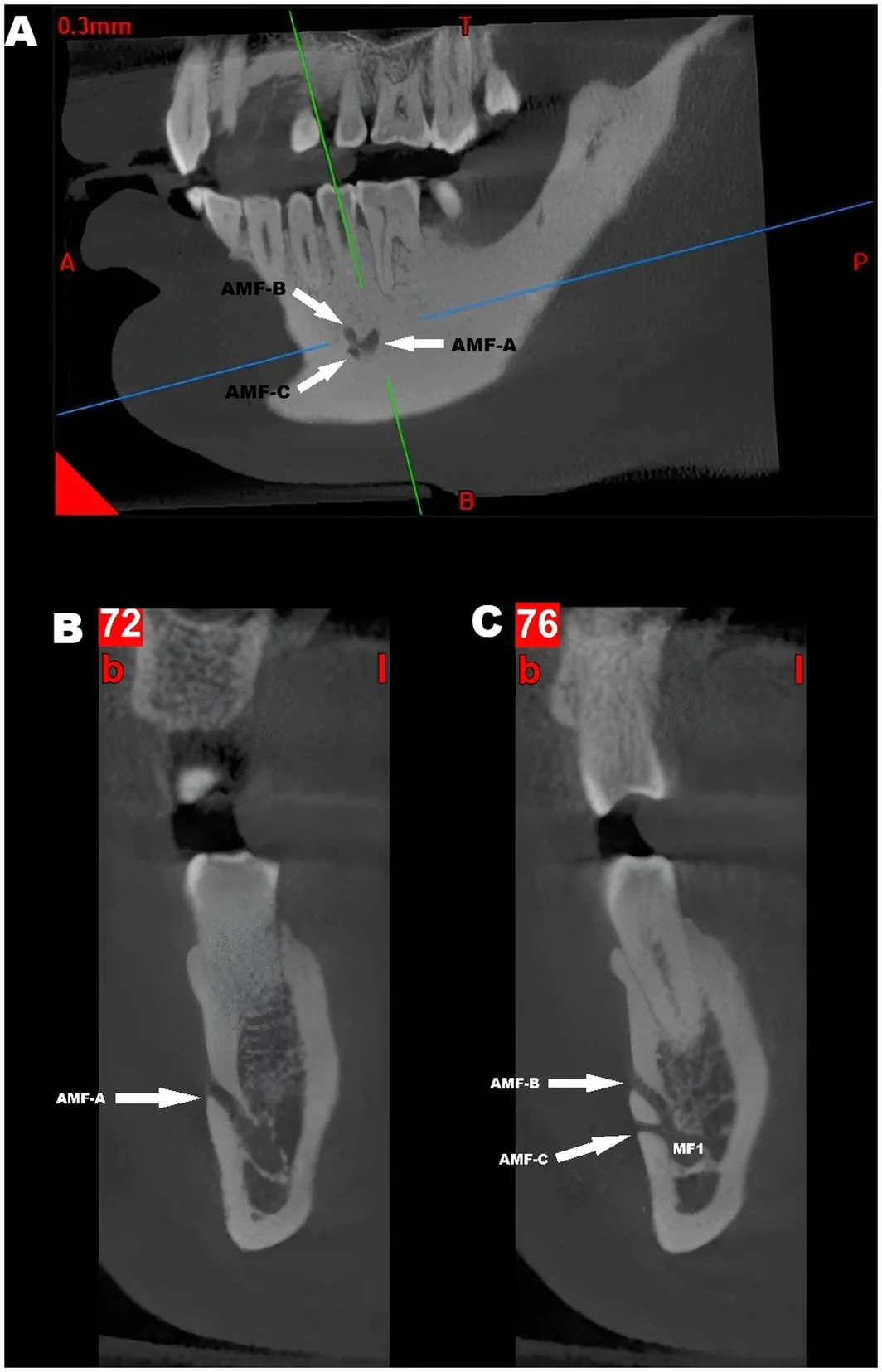
Radiographic views of the right side of the mandible. (A) Sagittal view showing three accessory mental foramina: AMF‐A, AMF‐B, AMF‐C. (B) Coronal view showing AMF‐A. (C) Coronal view showing AMF‐B and AMF‐C.

### 4.2 Left Mandibular Side (Two Foramina)

Left Primary Mental Foramen (MF2): Measuring 2.8 mm in diameter, located at the level between the second premolar and first molar apices, 9.0 mm superior to the inferior mandibular border (Figure 5A).

- Posterior Accessory Foramen (AMF‐D): A 1 mm opening situated 4 mm superior and slightly posterior to the primary foramen, located between the root apices of the first molar (Figures 5B and 6A,B).
- Anterior Accessory Foramen (AMF‐E): A 1 mm opening located right against the apex of the second premolar, representing the most anterior extent of the cluster (Figures 5B and 7A,B).

**FIGURE 5:**
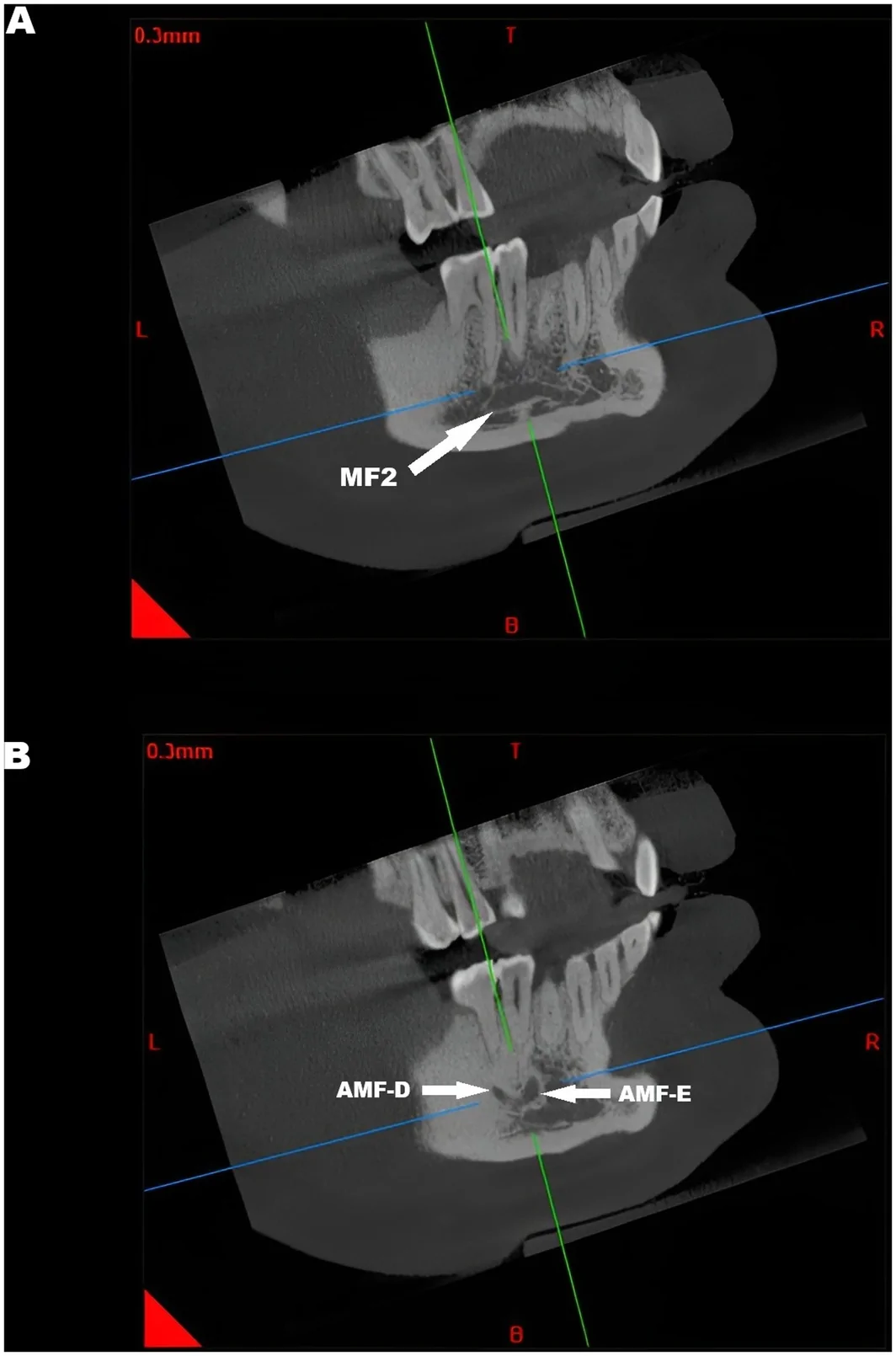
Sagittal radiographic view of the left mandible. (A) Showing the main mental foramen MF2. (B) Showing two accessory mental foramina AMF‐D and AMF‐E.

**FIGURE 6:**
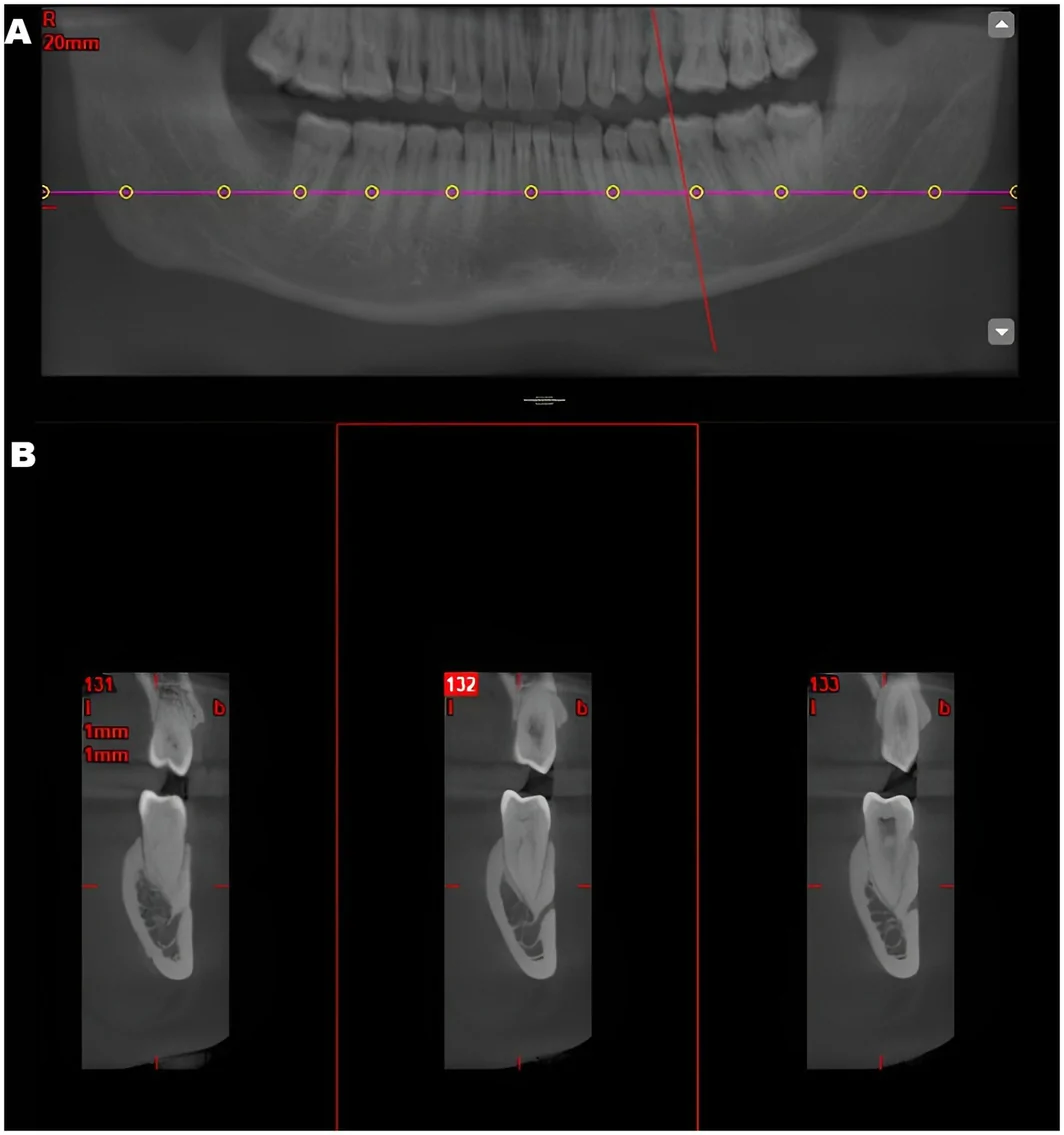
Radiographic views of AMF‐D. (A) Panoramic view. (B) Coronal view showing AMF‐D.

**FIGURE 7:**
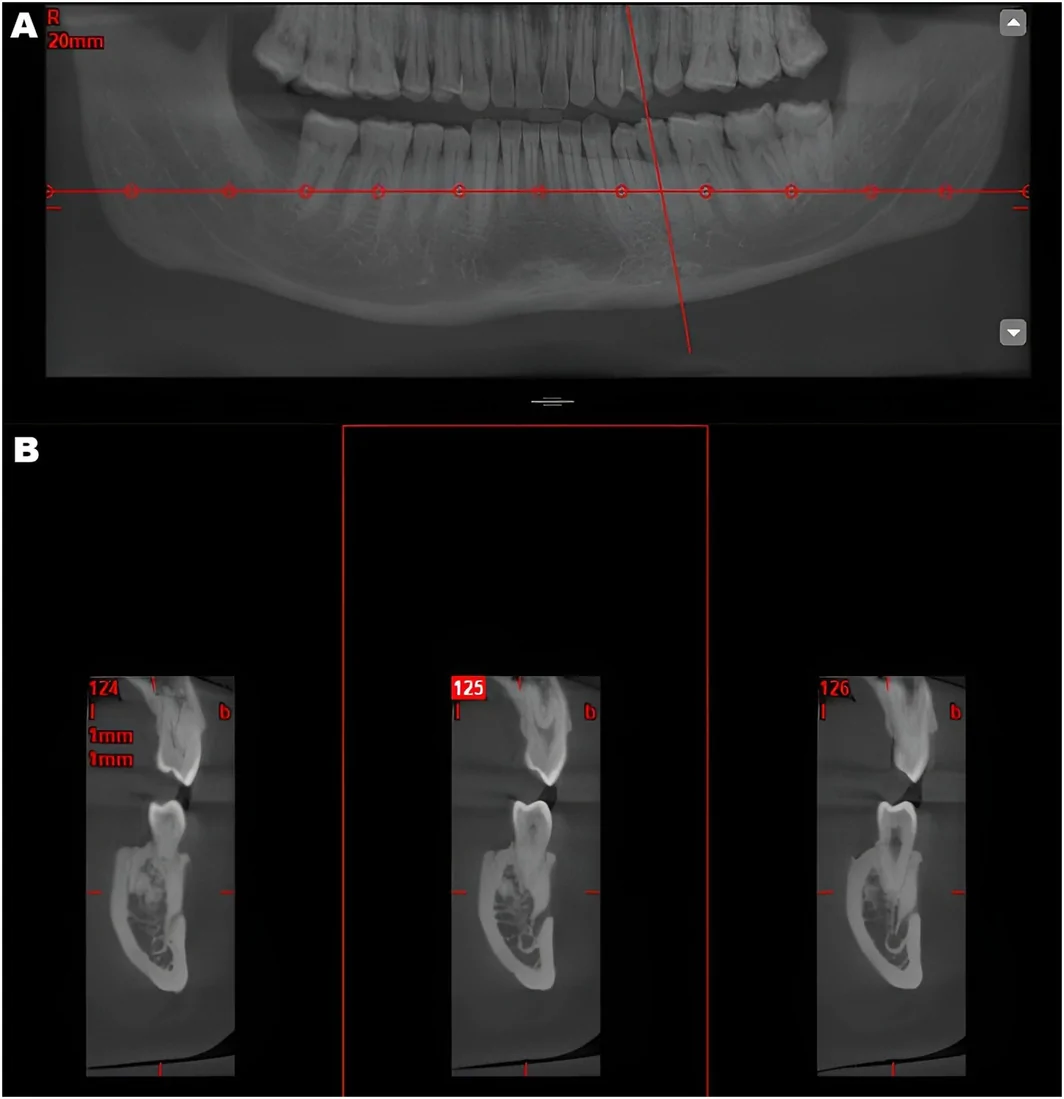
Radiographic views of AMF‐E. (A) Panoramic view. (B) Coronal view showing AMF‐E.

## 5 Treatment, Surgical Management & Clinical Outcome

Following the anatomical mapping provided by the CBCT scan, surgical extraction of the symptomatic lower right third molar was performed under local anesthesia. To ensure complete anesthesia despite potential accessory innervation, a standard inferior alveolar nerve block (IANB) was supplemented with local infiltration adjacent to the accessory exit points. Surgical extraction was completed smoothly without disturbing the neurovascular bundles in the premolar regions. The patient experienced an uneventful healing process. At the 2‐week and 3‐month postoperative follow‐up visits, soft tissue healing was complete, and neurosensory testing (light touch and two‐point discrimination) confirmed normal, intact sensation in the lower lip, labial gingiva, and chin bilaterally.

## 6 Discussion

The bilateral presence of accessory mental foramina yielding a total of five cortical exit points represents a remarkably rare anatomical presentation. While single accessory mental foramina are reported in approximately 2%–10% of individuals across various global populations, cases featuring three or more foramina account for < 1.2% of documented cases [5]. The identification of this quintuple arrangement underlines the extensive morphological heterogeneity of the mandibular neurovascular architecture. This pentagonal distribution suggests that the sensory supply to the mental region is fragmented into multiple smaller filaments, each exiting through its own osseous portal, which increases the surface area of the mandible that must be treated as “highly sensitive” to surgical trauma [6].

From an embryological perspective, this configuration stems from incomplete consolidation of nerve branches and ossification centers during the 12th week of intrauterine life. During early development, the mental nerve ramifies into multiple sensory fascicles. In typical development, these fascicles coalesce before cortical bone formation, forming a single osseous exit. When ossification occurs around premature neurovascular branches [7], discrete cortical micro‐channels are formed, resulting in multiple accessory foramina.

The clinical significance of identifying such variants lies in the challenge to the traditional “Safe Zone” concept. Clinicians often assume a single neurovascular bundle; however, failure to recognize accessory neurovascular bundles can lead to iatrogenic injury. In the context of implant surgery, the “anterior loop” of the mental nerve may be more complex and widespread in the presence of accessory foramina. Standard protocols suggesting a 2–3 mm safety margin from the primary mental foramen are insufficient here [8]. An implant placed between any of these five openings could cause direct nerve transection or indirect compression, leading to permanent paresthesia.

Furthermore, multiple foramina can lead to “escape” innervation, which complicates local anesthesia. If a clinician performs a local infiltration at the site of the primary foramen, branches exiting through the accessory foramina, particularly those located more superiorly or inferiorly, may not be fully anesthetized. This explains cases of “incomplete anesthesia” despite clinically accurate needle placement [9]. For surgical procedures involving mucoperiosteal flaps, such as periapical surgery or cyst enucleation, the risk of “accidental neurotmesis” is effectively quintupled. Standard vertical releasing incisions must be avoided or placed significantly further anteriorly to prevent transecting one of these accessory bundles [4].

## 7 Sex and Lateral Predilection

A critical question in clinical anatomy is whether accessory mental foramina display sex or side predilection. Literature analyses using large CBCT cohorts yield variable results. Regarding sex predilection, several population studies (e.g., Sisman et al., Naitoh et al.) [2, 10] report a slightly higher prevalence of accessory mental foramina in male individuals compared to females. This observation is often attributed to greater overall mandibular dimensions, increased bone density, and higher masticatory muscle forces driving cortical bone remodeling in males. However, other meta‐analyses indicate that while absolute dimensions of the primary foramen differ by sex, the occurrence rate of accessory foramina does not reach statistical significance between genders in all ethnic groups.

Regarding lateral distribution (right vs. left side), epidemiological data demonstrate no statistically significant side predilection overall, showing an approximately equal distribution between the right and left mandibular bodies. Nevertheless, unilateral occurrence remains far more common than bilateral involvement. Bilateral presentation of multiple accessory foramina, as demonstrated in our patient (three on the right, two on the left), represents an exceptionally low frequency within the literature, emphasizing the necessity of routine bilateral evaluation when an anomaly is identified on one side.

## 8 Clinical Implications

The clinical significance of identifying multiple foramina lies in avoiding iatrogenic nerve injury, preventing inadequate local anesthesia, and planning surgical procedures. In implantology, the traditional 2–3 mm safety margin from a single primary foramen is insufficient when accessory branches exit superiorly or anteriorly. Surgical flap design, particularly vertical releasing incisions in periapical or periodontal surgery, must accommodate accessory nerve filaments to prevent postoperative dysesthesia or paresthesia. Furthermore, “escape” innervation through accessory foramina explains why conventional local anesthetic blocks occasionally fail, necessitating supplementary infiltration around secondary foramina.

## 9 Diagnostic Role of CBCT & Radiation Stewardship

Conventional two‐dimensional radiographs (such as panoramic or periapical views) frequently fail to demonstrate AMFs due to anatomical overlap, radiopaque noise, and small canal diameters (< 1.0 mm) [10]. CBCT remains the gold standard for visual mapping. However, in keeping with ALARA/ALADA guidelines, 3D cross‐sectional imaging should be prescribed judiciously with localized fields of view and appropriate voxel sizes tailored to the clinical diagnostic requirement, rather than as a routine screening tool to allow clinicians to trace accessory canals back to their origin in the mandibular canal, distinguishing a true AMF from a nutrient canal or a pathological lesion [11]. In this case, only 3D rendering could accurately map the “fan‐like” divergence of the five branches.

Ultimately, this case underscores the necessity of a patient‐specific approach to surgical mapping and challenges the reliance on legacy anatomical landmarks. While textbook descriptions provide a foundational understanding of the “average” patient, they can lead to clinical complacency. As high‐resolution imaging becomes ubiquitously available, the nomenclature of “accessory” foramina may shift as we realize these structures represent a spectrum of normal variation that was simply invisible to less sensitive radiographic modalities [12]. This paradigm shift toward recognizing high‐frequency morphological variance is crucial for reducing neurosensory complications and refining the standards of care in oral and maxillofacial surgery.

## 10 Conclusion

This case documents a rare presentation of five mental foramina distributed bilaterally in a male patient. It underscores the importance of high‐resolution CBCT imaging under ALARA/ALADA radiation principles when complex anatomy is suspected and highlights the need for clinicians to remain vigilant regarding neurovascular variations to ensure safe surgical outcomes.

## Author Contributions

**Joul Kassis:** conceptualization, investigation, methodology, data curation, visualization, writing – review and editing, writing – original draft, validation, supervision, formal analysis.

## Funding

The author has nothing to report.

## Ethics Statement

Written informed consent from the patient was obtained according to journal guidelines.

## Conflicts of Interest

The author declares no conflicts of interest.

## Data Availability

The data that support the findings of this study are available on request from the corresponding author. The data are not publicly available due to privacy or ethical restrictions.
